# Preparation for birth and complication readiness: rural-urban disparities among pregnant women in communities in Enugu State, Nigeria

**DOI:** 10.11604/pamj.2022.42.310.33015

**Published:** 2022-08-25

**Authors:** Ifeoma Maureen Obionu, Miriam Ajuba, Emmanuel Nwabueze Aguwa

**Affiliations:** 1 Department of Community Medicine, University of Nigeria Teaching Hospital, Enugu State, Nigeria

**Keywords:** Birth preparedness, complication readiness, maternal deaths, danger signs, pregnancy

## Abstract

**Introduction:**

birth preparedness and complication readiness (BPCR) is a key component of safe motherhood programs. The study aimed to determine the rural-urban disparities in BPCR and its predictors among pregnant women.

**Methods:**

this study was a community-based comparative cross-sectional study carried out among 366 pregnant women living in rural and urban areas in Enugu State, Nigeria. A multistage sampling technique was used to select the participants. Data analysis was carried out using descriptive statistics and inferential statistics at a significant level of p < 0.05.

**Results:**

among the respondents, 213 (58.2%) had good knowledge of the components of BPCR. However, a significantly higher proportion of those in urban areas had better knowledge of these components than those in rural areas (p=0.01). Generally, there was a poor practice of BPCR among both groups of respondents. However, between both groups of respondents, good practice of BPCR was statistically significantly higher in respondents from urban areas 69 (37.7%) than those in rural areas 47 (25.7%) (X^2^=6.108, p=0.013). Several factors were found to be associated with good practice of BPCR among the respondents however, the only predictor of good practice of BPCR among the urban respondents was being aware of free maternal and child health services in the State while for the rural respondents, it was having an assisted delivery in the last stages of pregnancy.

**Conclusion:**

there are rural-urban disparities in BPCR. Most pregnant women are knowledgeable about its components but the majority do not practice it appropriately.

## Introduction

Pregnancy should be a normal physiological process that creates pleasure and anticipation for the woman as well the family and the community. However, the outcome is unpredictable, and a pregnant woman can have difficulties during the antenatal, intrapartum, or postnatal period, which can lead to the death of the mother or negatively affect the child [1]. The burden of maternal death is presently alarming; about 289,000 or more maternal deaths occurred worldwide in 2013 and 62% (179,000 maternal deaths) of these estimated deaths occurred in sub-Sahara Africa (SSA) alone [2]. There are several reasons these maternal deaths occur one of which is the inadequacy or lack of birth and emergency preparedness [3]. Indeed, the National Primary Health Care Development Agency (NPHCDA) April 2019 declared a state of emergency on maternal and child mortality due to reports that Nigeria was among the nations with the worst maternal mortality ratios [4]. In Nigeria, maternal mortality was said to be higher in rural when compared with urban areas and this could be because of the limited use of health services available to mothers and children, particularly in rural areas [5]. Even though BPCR is essential for advancing maternal and child health and several interventions have been carried out in recent times, there is a paucity of research on the recent possible disparities in rural and urban areas and how large these disparities may be in the South-Eastern part of Nigeria.

The authors hope to not only establish and/or confirm these disparities in BPCR, if any, between urban and rural areas in Enugu State, Southeast Nigeria, and factors influencing the disparities but to also further help to identify and develop interventions for solving this problem. Birth preparedness and complication readiness (BPCR) is an important component of safe motherhood initiatives intended to reduce delays around care-seeking, reaching and receiving care during birth, and promoting skilled care at delivery and in the early postnatal period [6]. Studies from countries like India, Nepal, and Nigeria have shown that BPCR has increased the level of knowledge of women about danger signs of pregnancy with a consequent improvement in their health-seeking behavior [6-8] therefore, encourages pregnant women to plan and prepare for birth during the antenatal period in case of unforeseen adverse events, thereby preventing possible death to mothers and their newborns and contributing to the progress towards attaining the Sustainable Development Goals (SDGs) [9]. Acknowledging the importance of BPCR, the World Health Organization (WHO) has recommended that all pregnant women should have a plan written down for birth and for handling unforeseen adverse events such as complications and emergencies that may take place while pregnant, during delivery, or in the early postnatal period, and should discuss and review this plan with a skilled attendant at each antenatal visit at least one month before the expected date of birth [10]. It has been noted that the absence of planning for the services of a skilled birth attendant and inadequate BPCR are known predictors of the delay in accessing skilled obstetric care [11].

Low levels of knowledge of obstetric danger signs and BPCR have been reported in several studies in Africa including Nigeria. A study carried out in 2015 in Enugu State in Southeast Nigeria to compare the rural and urban differences in the knowledge of women on important danger signs of pregnancy - a component of BPCR showed that a significantly higher percentage of pregnant women in rural areas 145 (53.7%) had good knowledge of danger signs when compared to urban areas 104 (38.5%) (p-value <0.001) [12]. Conversely, a study was done in 2014 in Anambra State, Southeast Nigeria, found no significant difference in the knowledge of birth preparedness in respondents in rural and urban areas [13]. These unusual findings may be due to an increase in safe motherhood interventions by the government in conjunction with some non-governmental organizations, which appears to be more pronounced in rural areas since in Nigeria, maternal mortality is higher in rural compared to urban areas [5,12]. Other studies have however shown varying results [14,15] a study in Aleta Wando District in Southern Ethiopia, showed that being an urban resident increased the likelihood of having good knowledge of danger signs of pregnancy [16] and in Lagos State, Nigeria, the overall level of adequate BPCR was quite low, although, was significantly higher in urban areas when compared with that of the rural areas [15]. The objective of this research is to provide information on the recent possible disparities in the knowledge and practice of BPCR and knowledge of danger signs in pregnancy among pregnant women living in rural and urban communities in Enugu State, Southeast Nigeria, and identify factors associated with the practice of birth preparedness and complication readiness in pregnant women living in rural and urban communities. Furthermore, the findings of this study will contribute credible information for health service providers, health stakeholders, and health managers to design effective BPCR interventions to reduce maternal and neonatal mortality rates in Nigeria. It is hoped that the results of our study will also serve as baseline information for future related research.

## Methods

**Study design and setting:** this was a community-based comparative cross-sectional study carried out between August 2020 and March 2021 among 366 pregnant women residing in rural and urban communities in Enugu State, Nigeria. Enugu State is located in the southeast geopolitical zone of Nigeria. There are seventeen local government areas in the State and four of these are largely urban.

**Study population and data collection:** the sample size was determined using the sample size estimation formula for comparing two proportions [16] and a prevalence of 53.7% and 38.5% [12] (proportion of women with good knowledge of danger signs in pregnancy in rural and urban areas of Enugu State respectively) from a similar study. This sample size was calculated using a standard normal deviation of 1.96 at a 95% confidence level and a power set at 80%. Only pregnant women who had lived in the selected communities for 6 months or more were allowed to participate in the study. A rural and an urban community were selected using a simple random sampling method. Four wards each were then selected from the rural and urban local government organizations (LGAs) by simple random sampling using the balloting method. The households were also selected using a simple random sampling method. In households where there was more than one eligible respondent, one respondent was selected by balloting for the interview. Data were collected using a pre-tested interviewer-administered semi-structured questionnaire and variables were adapted from the safe motherhood Johns Hopkins Program for the International Education in Gynaecology and Obstetrics Prototype questionnaire and other research works [9,12,17]. The questionnaire included demographic and obstetric information, and questions to assess the knowledge of obstetric danger signs and the components of BPCR. The overall time for completion of each questionnaire was about 20 minutes.

**Statistical analysis:** poor knowledge of obstetric danger signs was the ability to correctly mention less than three of the danger signs assessed, while good knowledge was the ability to correctly mention 3 or more danger signs. Women, who knew at least four of the seven BPCR components assessed, were classified as having good knowledge of BPCR, and those who knew less than four of the components were classified as having poor knowledge. To determine the practice of BPCR, the respondents were assessed for the presence of seven basic components of birth preparedness and complication readiness, and a woman was said to be well prepared if she reported at least four of the seven components and poorly prepared if less than four of the BPCR components were reported. Data was entered and analyzed using Epi Info version 6. Results are presented as frequencies and cross-tabulations. Chi-square test was used to test for significance of association between the independent variables (age, marital status, currently living with partner/spouse, main decision-maker in the household regarding health issues, respondents' education, education of spouse/partner, registered with health insurance scheme, gravidity, trimester of pregnancy, history of complication in a previous pregnancy, history of stillbirth in a previous pregnancy, registered for ante natal care (ANC), number of ANC visits, knowledge of the expected date of delivery, delivery type in last pregnancy, awareness of free maternal, child and neonatal care, walking distance from home to nearest health facility) and dependent variable (practice of BPCR) and multivariate logistic regression analysis was used to determine the factors that significantly predict the practice of BPCR among the respondents. Factors significant in bivariate analysis (Chi-square) were incorporated into the regression model. The logistic regression was used to adjust for confounding factors. The level of statistical significance was set at p< 0.05.

**Ethical consideration:** ethical approval for the study was received from the Health Research and Ethics Committee of the University of Nigeria Teaching Hospital, Ituku-Ozalla, and Enugu, and informed consent was obtained from all the pregnant women.

## Results

A total of 366 respondents participated in the study. These included 183 pregnant women in rural areas and 183 pregnant women in urban areas of Enugu State. All meet the eligibility criteria and completed the questionnaire. The mean age of the respondents was 29 years. Most of the respondents in both groups were multigravidas 245 (66.9%) and were in the second trimester of their index pregnancy 117(63.9) (Table 1). Table 2 shows the knowledge of danger signs in pregnancy among the respondents. The most common danger sign known by the respondents in both areas was bleeding from the vagina 264(72.1%) while swollen hands and feet were the least known danger sign respondents reported 100 (27.3%). There was no statistically significant difference between the two groups in the knowledge of any of the danger signs assessed. Both respondents from the rural and urban areas had good knowledge of BPCR 95 (51.9%) and 118 (64.5%) respectively. The most common component of BPCR known by the respondents was purchasing delivery kit items 248 (67.8%). A higher number of the respondents in the urban areas 69 (37.7%) knew that identification of a compatible blood donor was a component of BPCR when compared with their rural counterparts 41 (22.4%) and this difference was statistically significant (X^2^= 10.190, p=0.001) (Table 3).

**Table 1:** sociodemographic characteristics of respondents in rural and urban communities in Enugu State, Nigeria

Variable	Rural n=183	Urban (n=183)	Total (N=366)	χ^2^	P -value
	Freq (%)	Freq (%)	Freq (%)		
**Age (years)**					
17-24	33 (18.0)	21 (11.5)	54 (14.8)	3.155	0.207
25-34	136 (74.3)	146 (79.8)	282 (77.0)		
35-49	14 (7.7)	16 (8.7)	30 (8.2)		
**Marital status**					
Married	176 (96.2)	174 (95.1)	350 (95.6)	2.302f	0.512
Widowed	2 (1.1)	4 (2.2)	6 (1.6)		
Single	3 (1.6)	3 (1.6)	6 (1.6)		
Divorced/separated	2 (1.1)	2 (1.1)	4 (1.2)		
**Currently living with spouse/partner**					
Yes	177 (96.7)	174 (95.1)	351 (95.9)	0.626	0.429
No	6 (3.3)	9 (4.9)	15 (4.1)		
**Main decision maker in the household regarding health issues**					
Husband /partner	157 (85.8)	156 (85.2)	313 (85.5)	0.520	0.771
Self	8 (4.4)	6 (3.3)	14 (3.8)		
Others	18 (9.8)	21(11.5)	39 (10.7)		
**Highest level of education of respondents**					
None	35 (19.1)	19 (10.4)	54 (14.8)	6.933	0.074
Primary	53 (29.0)	51 (27.9)	104 (28.4)		
Secondary	67 (36.6)	74 (40.4)	141(38.5)		
Tertiary	28 (15.3)	39 (21.3)	67 (18.3)		
**Average monthly household income (Naira)**					
18000 or less	15 (8.2)	30 (16.4)	45 (12.3)	14.172	*0.003
18001-50000	30 (16.4)	22 (12.0)	52 (14.2)		
50001-82000	75 (41.0)	49 (26.8)	124 (33.9)		
>82000	63 (34.4)	82 (44.8)	145 (39.6)		
**Wealth index**					
Lowest quintile	12 (6.6)	7 (3.8)	19 (5.2)	12.156	*0.016
Second quintile	81(44.3)	59 (32.2)	140 (38.3)		
Middle quintile	65 (35.5)	70 (38.3)	135 (36.9)		
Fourth quintile	15 (8.2)	24 (13.1)	39 (10.7)		
Highest quintile	10 (5.5)	23 (12.6)	33 (9.0)		
**Highest level of education of spouse/partner**					
None	29 (16.3)	13 (7.4)	42 (11.9)	15.136	*0.002
Primary	55 (30.9)	35 (20.0)	9 (25.5)		
Secondary	67 (37.6)	86 (49.1)	153 (43.3)		
Tertiary	27 (15.2)	41(23.4)	68 (19.3)		

* Statistically significant (P-value < 0.05), χ^2^; Chi-square test, n; number of participants in each category, N; total number of participants

**Table 2:** knowledge of danger signs in pregnancy in respondents by study area

Variable	Rural	Urban	Total	χ^2^	P-value
	(n=183)	(n=183)	(N=366)		
	Freq (%)	Freq (%)	Freq (%)		
**Bleeding from the vagina**					
Know	134 (73.2)	130 (71.0)	264(72.1)	0.217	0.641
Did not know	49 (26.8)	53(29.0)	102 (27.9)		
**Liquor drainage before term**					
Did not know	99 (54.1)	87(47.5)	186 (50.8)	1.574	0.210
Know	84(45.9)	96 (52.5)	180 (49.2)		
**Foul-smelling vaginal discharge**					
Did not know	132(72.1)	126(68.9)	258 (70.5)	0.473	0.492
Know	51 (27.9)	57 (31.1)	108 (29.5)		
**Severe abdominal pains**					
Know	118 (64.5)	133 (72.7)	251 (68.5)	2.853	0.091
Did not know	65 (35.5)	50 (27.3)	115 (31.4)		
**Swollen hands and feet**					
Know	42 (30.0)	58 (31.7)	100 (27.3)	3.522	0.061
Did not know	141 (77.0)	125 (68.3)	266 (72.7)		
**Fits**					
Did not know	155(84.7)	150 (82.0)	305(83.3)	0.492	0.483
Know	28 (15.3)	33(18.0)	61(16.7)		
**Knowledge of danger signs in pregnancy**					
Good knowledge	122 (66.7)	135 (73.8)	25 (70.2)	2.208	0.137
Poor knowledge	61(33.3)	48 (26.2)	10 (29.8)		

χ^2^;Chi-square test, n; number of participants in each category, N; total number pf participants

**Table 3:** knowledge of birth preparedness and complication readiness components in respondents by study area

Variable	Rural (n=183) Freq (%)	Urban (n=183) Freq (%)	Total (N=366) Freq (%)	χ^2^	P- value
**Identification of compatible blood donor**					
Know	41 (22.4)	69 (37.7)	110 (30.1)	10.190	*0.001
Did not know	142 (77.6)	114 (62.3)	256 (69.9)		
**Transportation arrangement**					
Know	69 (37.7)	75 (41.0)	144 (39.3)	0.412	0.521
Did not know	114 (62.3)	108 (59.0)	222 (62.0)		
**Identification of a place for delivery**					
Know	104 (56.8)	124 (67.8)	228 (62.3)	4.653	*0.031
Did not know	79 (43.2)	59 (32.2)	138 (37.7)		
**Save money**					
Know	96 (52.5)	94 (51.4)	190 (51.9)	0.044	0.834
Did not know	87 (47.5)	89 (48.6)	176 (48.1)		
**Identification of a skilled birth attendant for delivery**					
Know	87 (47.5)	104 (56.8)	191 (52.2)	3.165	0.075
Did not know	96 (52.5)	79 (43.2)	175 (47.8)		
**Availability of birth companion**					
Know	63 (34.4)	70 (38.3)	133 (36.3)	0.579	0.447
Did not know	120 (65.6)	113 (61.7)	233 (67.3)		
**Purchasing delivery kit items**					
Know	119 (65.0)	129 (70.5)	248 (67.8)	1.251	0.263
Did not know	64 (35.0)	54 (39.5)	118 (32.2)		
**Knowledge of BPCR**					
Good knowledge	88 (48.1)	65 (35.5)	153 (41.8)	5.921	*0.015
Poor knowledge	95 (51.9)	118 (64.5)	213 (58.2)		

* Statistically significant (P-value < 0.05), χ^2^;Chi-square test, n; number of participants in each category,N; total number of participants

Few of the respondents 116(31.7%) had good practice of BPCR. Between both groups, good practice of BPCR was statistically significantly higher in respondents from the urban areas 69(37.7%) than those in the rural areas 47(25.7%) (X^2^=6.108, p=0.013) (Table 4). Among the rural respondents, factors associated with the poor practice of BPCR were: being aged between 17-34 years, households where the main decision-maker on health issues were partners/spouses, being a multigravida, having no previous history of stillbirth, women with normal deliveries in their last pregnancy, women who were not aware of the free maternal and child health services available in the State, and women who lived less than 30 minutes away from a health facility. While in the urban respondents, factors associated with the poor practice of BPCR were: households, where the main decision-maker on health issues were partners/spouses, being a multigravida, no previous history of stillbirth, women with normal deliveries in their last pregnancy, women in their second trimester of pregnancy, women who knew their employment development department (EDD) and women who were not aware of the free maternal and child health services available in the State (Table 5). Following regression analysis, the only predictor of BPCR in rural respondents found, was delivery type in the last pregnancy (AOR=5.015, CI=1.437-1.612), while for the urban respondents; it was awareness of free maternal and child health services in the State (AOR=1.252, CI=0.067-0.941).

**Table 4:** practice of birth preparedness and complication readiness components by respondents by study area

Variable	Rural (n=183) Freq (%)	Urban (n=183) Freq (%)	Total (N=183) Freq (%)	χ^2^	P-value
**Identified compatible blood donor**					
Yes	32 (17.5)	41 (22.4)	73 (19.9)	1.386	0.239
No	151 (82.5)	142 (77.6)	293 (80.1)		
**Made transportation arrangements to place of delivery or in case of emergency**					
Yes	42(23.0)	43(23.5)	85 (23.2)	0.015	0.901
No	141 (77.0)	140 (76.5)	281 (76.8)		
**Identified a place for delivery**					
Yes	66 (36.1)	95 (51.9)	161 (44.0)	9.326	*0.002
No	117(63.9)	88 (48.1)	205 (56.0)		
**Saved money**					
Yes	77 (42.1)	66 (36.1)	143 (39.1)	1.389	0.239
No	106 (57.9)	117 (63.9)	223 (60.9)		
**Identified a skilled birth attendant for delivery**					
Yes	54 (29.5)	81 (44.3)	135 (36.9)	8.556	*0.003
No	129 (70.5)	102 (55.7)	231 (63.1)		
**Made arrangement for a birth companion**					
Yes	43 (23.5)	44 (24.0)	87 (23.8)	0.015	0.902
No	140 (76.5)	139 (76.0)	279 (76.2)		
**Started purchasing delivery kit items**					
Yes	73 (39.9)	77 (42.1)	150 (41.0)	0.181	0.671
No	110 (60.1)	106 (57.9)	216 (59.0)		
**Practice of BPCR**					
Good practice	47 (25.7)	69 (37.7)	116 (31.7)	6.108	*0.013
Poor practice	136 (74.3)	114 (62.3)	250 (68.3)		

* Statistically significant (P-value < 0.05), χ^2^;Chi-square test, n; number of participants in each category, N: total number of participants

**Table 5:** factors associated with the practice of birth preparedness and complication readiness in respondents within study areas

Variable	Rural n=183				Urban n=183			
	Good practice of BPCR	Poor practice of BPCR	χ^2^	P-value	Good practice of BPCR	Poor practice of BPCR	χ^2^	P-value
	Freq (%)	Freq (%)			Freq (%)	Freq (%)		
**Age**								
17-34	40 (23.7)	129 (76.3)	4.697	*0.030	58 (34.7)	109 (65.3)	7.194	0.007
35-49	7 (50.0)	7 (50.0)			11 (68.7)	5 (31.3)		
**Main decision-maker in the household regarding health issues**								
Self	4(50.0)	4 (50.0)	8.936f	*0.011	3 (50.0)	3 (50.0)	11.131f	*0.004
Partner/spouse	43 (27.4)	114 (72.6)			65 (41.7)	91 (58.3)		
Others	0(0)	18(100.0)			1 (4.8)	20 (95.2)		
**Education of spouse/partner**								
Formal education	42 (28.2)	107 (71.8)	1.497	0.221	65 (39.9)	98 (60.1)	5.321f	*0.021
No formal education	5 (17.2)	24 (82.8)			1 (7.7)	12 (92.3)		
**Gravidity**								
Primigravida	8 (14.3)	48 (85.7)	5.492	*0.019	18 (27.7)	47 (72.3)	4.017	*0.045
Multigravida	39 (30.7)	88 (69.3)			51 (43.2)	67 (56.8)		
**Trimester of pregnancy**								
First trimester	8 (33.3)	16 (66.7)	5.406	0.067	10 (37.0)	17 (63.0)	5.692	*0.058
Second trimester	21 (19.6)	86 (80.4)			38 (32.5)	79 (67.5)		
Third trimester	18 (35.3)	33 (64.7)			21 (53.8)	18 (46.2)		
**History of stillbirth in previous pregnancy**								
Yes	12 (54.5)	10 (45.5)	8.323	*0.004	12 (85.7)	2 (14.3)	14.584f	*0.000
No	25 (23.8)	80 (76.2)			34 (32.7)	70 (67.3)		
**Knowledge of EDD**								
Yes	21 (28.4)	53 (71.6)	0.473	0.492	53 (45.7)	63 (54.3)	8.600	*0.003
No	26 (23.9)	83 (76.1)			16 (23.9)	51(76.1)		
**Delivery type in last pregnancy**								
Normal delivery	29 (27.6)	76 (72.4)	4.223	*0.040	38 (40.9)	55 (59.1)	4.251	*0.039
Assisted delivery	10 (45.5)	12 (54.5)			13 (52.0)	12 (48.0)		
**Awareness of free maternal, child and neonatal care**								
Yes	30(37.0)	51 (63.0)	9.815	*0.002	44 (49.4)	45 (50.6)	10.155	*0.001
No	17 (16.7)	85 (83.3)			25 (26.6)	69 (73.4)		
**Walking distance from home to nearest health facility**								
<30 minutes	28 (25.0)	84 ( 75.0)	*0.000	0.994	39 (35.8)	70 (64.2)	0.425	0.514
>30 minutes	19 (26.7)	52 (73.3)			30 (40.5)	44 (59.5)		

* =Statistically significant (P-value < 0.05), χ^2^;Chi-square test, n; number of participants in each category

## Discussion

Good knowledge of BPCR in the respondents was slightly above average and statistically significantly higher in respondents from the urban communities 118 (64.5%) when compared with the respondents from the rural communities 95 (51.9%). This corresponds with findings from a study in Anambra State Southeast Nigeria where slightly more than half (54.5%) of the respondents in the urban, compared to those in the rural (50.4%) had a fair knowledge of birth preparedness (BP) (p= 0.109) [13]. Also, more respondents in urban (62.1%) than in rural (59.5%) had poor knowledge of complication readiness (CR) (p=0.005). These rural-urban differences could be because of differences in some other socio-demographic and socio-economic characteristics between the two groups. In addition, as is the case in this study, a statistically significant difference was seen in registration for antenatal care (ANC) as more of the respondents in the urban areas than those in the rural areas had registered for ANC. It is a fact that health education activities are carried out at health facilities. Therefore, the respondents who had registered for ANC were likely to have received some health education regarding BPCR. This study also found that the components of BPCR that respondents were least knowledgeable about were similar in both rural and urban respondents, namely: identification of a compatible blood donor in case of an emergency, transportation arrangements, and availability of a birth companion. These were similar findings in Ghana [18] but different in Nepal [19] and Tanzania [20] where high levels of knowledge of BPCR components (94.4% and 89.3% respectively) were noted for fund arrangement while 93.6% and 92.3% had made transportation arrangements in Nepal and Tanzania respectively. The discrepancy may be due to regional differences in socioeconomic factors and infrastructural facilities. There was also a statistically significant difference between the knowledge of the urban respondents and rural respondents in the identification of a blood bank or blood donor and identification of a health facility for delivery as the urban respondents were more knowledgeable in these two components of BPCR.

More than half of the respondents in rural 122 (66.7%) and urban 135 (73.8%) communities were aware of the danger signs of pregnancy but the difference between both groups was not statistically significant. The most common danger signs spontaneously mentioned by both the rural and urban respondents was bleeding from the vagina 264 (72.1%). Similarly, in Southwest Nigeria, [12] knowledge of obstetric danger signs during pregnancy was also good among the rural and urban respondents and no statistically significant difference was found between the two groups. But this finding differed from another similar study in Southeast Nigeria [12] where a significantly higher proportion of rural respondents were better aware of the danger signs of pregnancy. However, this could have been attributed to the heightened community-based enlightenment programs of women groups on the danger signs of pregnancy carried out in the study area at the time of the research. Respondents in both rural and urban areas were least aware of foul-smelling vaginal discharge, swollen hands and feet, and fits (seizures) as danger signs in pregnancy. This also correlated with findings in a study in Abeshige District Ethiopia [21]. In Southeast Nigeria, similar findings were also observed where only 114 (42.2%) of urban and 130 (48.1%) of rural respondents were aware that foul-smelling vaginal discharge was a dangerous sign in pregnancy while 52(19.3%) of urban respondents and 72 (26.7%) of rural respondents were aware of fits and severe headaches as danger signs in pregnancy [12].

The practice of BPCR in both the rural and urban respondents in this study was found to be quite poor 69 (37.7%) and 47 (25.7%) respectively and this is similar to findings in different regions of Nigeria and other developing countries. In Southwest Nigeria, 40.3% of the respondents were well prepared for birth and its complications [22], and also, in Kano State in Northern Nigeria, 39.6% were well prepared [23]. In other developing countries, preparedness level was also found to be poor irrespective of region. For example, in Ethiopia, only 30.0% were well prepared for birth and its complications [24], in Rwanda, only 22.3% [25] and in Uganda, 35.0% [26] were well prepared. However, on the contrary, in Sokoto in Northern Nigeria, [27] a high level of BPCR was reported at 375 (92.0%) although this may be attributed to the fact that this study was carried out in an ANC clinic in a tertiary facility and majority of the respondents 322 (91.2%) resided in urban areas. There was a statistically significant difference (p=0.013) in the findings of this study when rural and urban respondents were compared. It was noted that the urban respondents were more prepared than the rural respondents. The result from this study may be because a statistically significant proportion (p=0.002) of urban respondents when compared to the rural were registered for ANC and this was a factor found to be associated with being well prepared for birth. Similarly, this was also a finding in Ethiopia, [28] and in Kenya [29].

In respondents in both the rural and urban areas, most had not identified a compatible blood donor, made transportation arrangements, and arranged for a birth companion. This may be because they were also the same components that the respondents were generally least knowledgeable about. In Ile-Ife South West Nigeria [30], only 45 (11.3%) had identified a potential blood donor and 249 (62.3%) had made transportation arrangements. Likewise, in Edo State Nigeria [31], only 10 (4.3%) and in Ethiopia only 30.2% had identified a potential blood donor [32]. This could be because their previous pregnancies may not have required any blood transfusion and most women believe pregnancy is a normal condition, and therefore, a critical situation requiring blood transfusion is unlikely to occur during pregnancy or labor. Studies [16,33] from other developing countries have also found that pregnant women in rural areas were less likely to be birth-prepared and complication ready when compared to women in urban settlements [34]. While age as a factor associated with BPCR has been shown to differ across regions, a study in Ethiopia [35] found that being in a younger age group of 21-25 was positively associated with BPCR, while age was not found to be associated with BPCR at all in Ghana and Addis Abba Ethiopia [24,36]. Although the finding from this study could be explained to mean that the older respondents were more likely to have had more children and (also had more experience) therefore are likely to be better prepared for birth than respondents in the younger age group. In rural respondents, older respondents between 35-49 years and being the main decision-maker in the household regarding health issues were socio-demographic characteristics found to be associated with BPCR while for the urban respondents, only being the main decision-maker in the household regarding health issues was associated with BPCR. More opportunities for future research can be directed toward assessing other socio-demographic variables which may be associated with BPCR.

Other factors also found in Delta State Nigeria to influence BPCR positively [37] include the history of obstetric complications and higher numbers of childbirth. These women were more likely to prepare for child delivery and were ready for possible complications. In this study, multiparity was also found to be a factor associated with BPCR in both rural and urban respondents. These were also similar findings in Nepal [38] where women were about fifteen times more likely to be prepared for later pregnancies than their first one and a study from North Ethiopia [14] reported that women with a parity range of two to four were more likely to prepare for birth and its complication than primiparous women. This can be because during previous pregnancies they may have received information about BPCR. Having a history of the previous stillbirth/neonatal death or having an assisted delivery in the last pregnancy also led to better preparedness in both the rural and urban respondents in this study as well as in studies in Ahmedabad city, India [39] and Northern Ethiopia [40] and this is due to the fear of the reoccurrence of these issues in affected individuals. Pregnant women in urban areas who knew their EDD were found to be better prepared which was a similar occurrence in India [41] and maybe because respondents' prior knowledge of their delivery date may have led them to be better prepared in advance to avoid any complications. However, being in a later trimester of pregnancy and knowledge of EDD were not statistically significant factors associated with BPCR in the respondents in the rural areas this is probably because few respondents in the rural areas knew their EDD.

In the rural and urban respondents, awareness of free maternal, child, and neonatal care services available in the State was found to lead to better preparedness in this study as these free services may have increased utilization of health services and more exposure to health information thus increasing good practice of BPCR and better health-seeking behavior. Due to this finding, activities to improve awareness of these free services must be put in place. Among the respondents in the rural areas, after adjusting for confounders, only having an assisted delivery in the last pregnancy was found to be a factor associated with BPCR and this may be because respondents may have experienced the lack of basic and comprehensive emergency obstetric care services in rural areas [42] which might have led to an increased fear of loss of their lives and that of their unborn baby. After adjusting for confounders in the factors associated with BPCR in the study participants in the urban areas, only being aware of the free maternal child and neonatal care services in the State was an independent factor associated with BPCR and this can be attributed to the fact that many rural women are not even aware of the free maternal and child program and ignorance persists among the rural women about their health needs and available solutions [43]. Furthermore, evidence has shown that the availability of free maternal and child health services has enhanced the utilization of health services [44] and also has led to a tremendous increase in the uptake of antenatal booking and hospital delivery in the Enugu Metropolis.

**Limitation:** this study was that study relied on self-reports from the respondents but this was mitigated by ensuring anonymity and strict confidentiality of the participant's responses and conducting interviews privately. The terms “poor knowledge” and “good knowledge” refer to the authors´ assessment of knowledge of obstetric danger signs in pregnancy among the respondents. It may be difficult to generalize the study findings to pregnant women outside the southeastern part of Nigeria because cultural diversity may vary based on residence.

## Conclusion

Findings from this study have shown that although most pregnant women in the rural and urban communities are knowledgeable about the components of BPCR, the majority still do not practice it appropriately and rural-urban differences regarding knowledge and practice of BPCR still exist in the South-Eastern part of Nigeria. Therefore, strategies already put in place to address these issues should be evaluated to improve their effectiveness.

### What is known about this topic

- Controversies regarding rural-urban disparities in maternal health care in South-Eastern part of Nigeria;
- Low levels of knowledge of obstetric danger signs and BPCR have been reported in several studies in Africa including Nigeria.

### What this study adds

- This study provides information on the recent possible disparities in knowledge and practice of BPCR, and knowledge of danger signs in pregnancy among pregnant women living in rural and urban communities, and how large these disparities are likely to be in the South-Eastern part of Nigeria;
- Identification of factors associated with and predictors of the practice of birth preparedness and complication readiness in pregnant.
